# Differential microRNA Profile in Operational Tolerance: A Potential Role in Favoring Cell Survival

**DOI:** 10.3389/fimmu.2019.00740

**Published:** 2019-04-25

**Authors:** Amanda Cabral, Darlan da Silva Cândido, Sandra Maria Monteiro, Francine Lemos, David Saitovitch, Irene L. Noronha, Letícia Ferreira Alves, Murilo Vieira Geraldo, Jorge Kalil, Edecio Cunha-Neto, Ludmila Rodrigues Pinto Ferreira, Verônica Coelho

**Affiliations:** ^1^Laboratório de Imunologia, Instituto do Coração (InCor), Faculdade de Medicina, Universidade de São Paulo, São Paulo, Brazil; ^2^Instituto de Investigação em Imunologia – Instituto Nacional de Ciências e Tecnologia (iii-INCT), São Paulo, Brazil; ^3^Serviço de Transplante Renal, Faculdade de Medicina, Universidade de São Paulo, São Paulo, Brazil; ^4^Divisão de Nefrologia, Hospital São Lucas, Pontifícia Universidade Católica do Rio Grande do Sul, Porto Alegre, Brazil; ^5^Laboratório de Nefrologia Celular, Genética e Molecular, Disciplina de Nefrologia, Faculdade de Medicina, Universidade de São Paulo, São Paulo, Brazil; ^6^Departamento de Biologia Estrutural e Funcional, Instituto de Biologia, Universidade Estadual de Campinas, São Paulo, Brazil; ^7^Laboratório de Histocompatibilidade e Imunologia Celular, LIM-19, Faculdade de Medicina, Universidade de São Paulo, São Paulo, Brazil

**Keywords:** kidney transplantation, operational tolerance, immunoregulation, chronic rejection, microRNAs, cell death, epigenetics

## Abstract

**Background:** Operational tolerance (OT) is a state of graft functional stability that occurs after at least 1 year of immunosuppressant withdrawal. MicroRNAs (*microRNA*) are small non-coding RNAs that downregulate messenger RNA/protein expression of innumerous molecules and are critical for homeostasis. We investigated whether OT in kidney transplantation displays a differential microRNA profile, which would suggest that microRNAs participate in Operational Tolerance mechanisms, and may reveal potential molecular pathways.

**Methods:** We first compared serum *microRNA* in OT (*n* = 8) with chronic rejection (CR) (*n* = 5) and healthy individuals (HI) (*n* = 5), using a 768-*microRNA* qPCR-panel. We used the Thermo Fisher Cloud computing platform to compare the levels of *microRNA*s in the OT group in relation to the other study groups. We performed validation experiments for *miR-885-5p*, by q-PCR, in a larger number of study subjects (OT = 8, CR = 12, HI = 12), as individual samples.

**Results:** We detected a differential *microRNA* profile in OT vs. its opposing clinical outcome—CR—suggesting that microRNAs may integrate transplantation tolerance mechanisms. Some miRNAs were detected at higher levels in OT: *miR-885-5p, miR-331-3p*, miR-27a-5p *vs*. CR; others, we found at lower levels: miR-1233-3p, miR-572, miR-638, miR-1260a. Considering highly predicted/experimentally demonstrated targets of these miRNAs, bioinformatics analysis revealed that the granzyme B, and death receptor pathways are dominant, suggesting that cell death regulation integrates transplantation tolerance mechanisms. We confirmed higher *miR-885-5p* levels in OT vs. CR, and vs. HI, in a larger number of subjects.

**Conclusions:** We propose that epigenetics mechanisms involving microRNAs may integrate human transplantation tolerance mechanisms, and regulate key members of the cell death/survival signaling. miR-885-5p could favor cell survival in OT by diminishing the levels of CRADD/RAIDD and CASP3. Nonetheless, given the nature of any complex phenomenon in humans, only cumulative data will help to determine whether this microRNA differential profile may be related to the cause or consequence of operational tolerance.

## Introduction

Despite its clinical success, allotransplantation still faces important challenges, such as preventing/treating chronic rejection (CR) and inducing immunotolerance. Nevertheless, a special group of transplant patients do not reject after stopping immunosuppressants, and preserve their immune competence. This phenomenon is called Operational Tolerance (OT). Studies on OT have mostly focused on the search for reliable OT biomarkers and determining potential underlying mechanisms (1–3). Several differential immunologic features have been reported for OT after renal transplantation, by our group and others, including the preservation of regulatory T (4, 5) and B cell (6, 7) numbers, contrasting with their loss in CR. Multiple mechanisms are believed to be involved in operational tolerance, but epigenetic mechanisms such as those exerted by microRNAs have only scarcely been investigated.

MicroRNAs are short non-coding RNAs that are 22 to 24 nucleotides in length (8). MicroRNAs may bind to target messenger RNAs and alter their stability leading to degradation and decreased/blocked protein translation (9). MicroRNAs regulate a vast array of molecules, including immune molecules (10), and affect basic cellular processes, such as cell differentiation and cell death. The evolutionary conservation of many sequences of microRNAs reveals their biological relevance and the critical role of microRNAs and epigenetics in homeostasis (11, 12). MicroRNAs are stable in body fluids and tissues, including serum (11, 12). They can be packaged in microvesicles and/or exosomes, or transported by RNA-binding proteins (Argonaute 2 complexes) and lipoproteins, conferring protection from degradation (13). MicroRNAs can be captured by cells, alter their transcriptional program and affect their functional activity (14, 15).

Different intracellular microRNA profiles have been associated with rejection in both humans (16) and in experimental models (17). However, it is still unclear whether they play a role in the mechanisms of rejection. In the only report on microRNAs in renal OT, 8 of 374 microRNAs studied were differentially expressed in the peripheral blood mononuclear cells (PBMC) of OT subjects, compared to stable patients (under immunosuppressants, without rejection), and miR-142-3p was expressed at a higher level in purified B cells in OT (18). Bioinformatics analysis revealed nearly 1,000 genes as potential targets for miR-142-3p, many related to immunologic activities, such as B cell activation and cellular communication. However, the lack of a comparison with chronic rejection limits the interpretations of this study regarding the potential mechanisms that contribute to these opposing clinical outcomes. Nevertheless, the comparison of the microRNA profile of OT and CR in liver transplantation showed higher expression of miR-95, miR-24, miR-31, miR-146a, and miR-155 in the blood of OT subjects compared to non-tolerant individuals who experienced cellular rejection following immunosuppressant withdrawal (19). These authors suggest that these microRNAs may play a role in transplant tolerance.

We investigated whether operational tolerance in renal transplantation presents a differential profile of serum microRNAs, which would suggest that these microRNAs participate in the mechanisms of human transplantation tolerance. We used a panel for 768 microRNAs and found a differential profile of seven microRNAs that primarily regulate targets involved in cell death and whose levels discriminate OT from CR. Validation experiments using *miR-885-5p* (in a larger number of study subjects) confirmed that there were higher levels in OT compared to both CR and healthy individuals. Because *miR-885-5p* regulates critical targets in the death pathways that include CRADD/RAIDD and the experimentally confirmed target caspase 3 (*CASP3)* (20), our data suggest that epigenetic mechanisms involving *miR-885-5p* and other microRNAs may have a relevant role in promoting cell survival in OT, and thereby, may integrate the network of mechanisms underlying human transplantation tolerance. Nevertheless, as in any complex biological phenomenon in humans, cumulative data is necessary to determine if this microRNA differential profile is related to the cause, or is a consequence of operational tolerance.

## Materials and Methods

### Experimental Design

We first compared the profile of 768 microRNAs serum levels in OT (Operational tolerance) and in CR (Chronic Rejection), which are two opposing clinical outcomes. We used the microRNAs of the *OT-CR differential profile* to identify potential targets and significant pathways in order to raise hypotheses regarding their potential differential roles in OT vs. CR. Finally, we selected *miR-885-5p* to validate the levels in a larger number of subjects. We then compared microRNA levels in OT vs. healthy individuals (HI). This comparison is critical to determine whether the levels of the microRNAs differentially detected in OT vs. CR (*OT-CR differential profile*) were increased, decreased or preserved in relation to the physiological state. All serum samples were analyzed individually.

### Study Groups

Renal transplant recipients from different medical centers in Brazil (Hospital das Clinicas da Universidade de São Paulo-SP, Unidade de Nefrologia da Pontifícia Universidade Católica do Rio Grande do Sul-RS, Hospital Beneficencia Portuguesa -SP and Hospital de Londrina-PR were enrolled in the *Brazilian Multicenter Study on Operational Tolerance*, coordinated at Instituto do coração da Universidade de São Paulo (USP). This study was approved by ethic committee at the School of Medicine-USP (CAPPesq-Ethics Committee for Research Projects; 0476/08 and 1097/05). All study subjects signed an informed consent agreement.

All included individuals had more than 1 year of time since their transplantation and were distributed in the following groups: (i) Operational Tolerance (OT, *n* = 8): Stable graft function without immunosuppressive medications for at least 1 year (mean serum creatinine: 1.2 mg/dl). (ii) Chronic rejection (CR, *n* = 12): individuals on standard immunosuppression, with progressive renal failure and histological diagnosis of chronic T cell or antibody mediated rejection according to Banff criteria (21) and (iii) Healthy individuals (HI, *n* = 12): healthy kidney donors with normal kidney function. In the chronic rejection group, four subjects had histological diagnosis of antibody mediated chronic rejection and eight subjects had chronic T cell mediated rejection. The mean serum creatinine in this group was 1.93 mg/dl (shown in Table 1).

**Table 1:** Demographic and clinical data of study subjects.

**Study Subjects**	**OT 03**	**OT 08**	**OT 10**	**OT 16**	**OT 62**	**OT 75**	**OT 90**	**OT 91**	**OT (*n* = 8)**	**CR (*n* = 12)**	**HI (*n* = 12)**	**p value OT x CR**	***p*-value OT x HI**
Age (Years)	55	42	42	48	32	37	31	57	43 (32–57)	45 (28–59)	45 (29–61)	*p* = 0.674^2^	*p* = 0.633^2^
Sex (F, M)	M	M	F	F	M	M	F	M	*F* = 3; *M* = 5	*F* = 2; *M* = 10	*F* = 6; *M* = 6	*p* = 0.347^1^	*p* = 0.669^1^
Transplantation number	1	2	2	1	1	2	1	1	1 (*n* = 5); 2 (*n* = 3)	1 (*n* = 2); 2 (*n* = 10)	NA	*p* = 0.668^2^	NA
Time of transplantation (years)	27	5	12	28	17	6	11	31	17 (5–28)	7 (1–13)	NA	*p* = 0.018^*^^2^	NA
HLA (A, B, DR) mismatches	3	4	0	0	3	1	1	6	6 (*n* = 1); 4 (*n* = 1); 3 (*n* = 2); 1 (*n* = 1); 0 (*n* = 2)	6 (*n* = 4); 5 (*n* = 3); 3 (*n* = 4); 1 (*n* = 2);	NA	*p* = 0.012^*^^2^	NA
Donor (Live/ Deceased)	Live	Deceased	Live	Live	Live	Deceased	Live	Live	Live (n = 6) Dead (*n* = 2)	Live (n = 4) Dead (*n* = 8)	NA	*p* = 0.169^1^	NA
Creatinine	1.15	1.62	0.89	0.74	1.71	1.1	1.4	1.3	1.23 (0.89–1.71)	1.93 (1.3–4.4)	NA	*p* = 0.014^*^^3^	NA
Immunosuppressive time free (years)	7	2	2	8	4	4	2	30	7 (2–30)	NA	NA	NA	NA
Immunosuppressive used	Aza, Pred	Pred Tacro MMF	Cya Aza Pred	Pred Aza	Cya Aza Pred	Tacr Pred MMF Eve	TacrMPS Pred	Aza Pred	Pred (*n* = 8) Tacro (*n* = 3) MMF (*n* = 2) MPS (*n* = 1) Cya (*n* = 2) Aza (*n* = 5) Eve (*n* = 1)	Pred (*n* = 1) Tacro (n8) MMF (*n* = 3) MPS (*n* = 6) Cya (*n* = 1) Aza (= 1) Eve(*n* = 1)	NA	NA	NA

*The ordinal variables were initially analyzed by the Shapiro-Wilk test to verify normality*.

1 Chi-square test;

2 t test;

3 *Mann-Whitney*.

The numerical data are represented as mean. The minimum and maximum values of the age of each group, transplant time, number of HLA disparities, donor type and creatinine are shown in parenthesis. The

* *p < 0.005. Operational Tolerance (OT) n = 8; Chronic Rejection (CR) n = 12; Healthy Individuals (HI) n = 12. NA, Not applicable; F, Female; M, Male; Pred, prednisone; Tacro, tacrolimus; MMF, mycophenolate mofetil; MPS, mycophenolate sodium, Cya, cyclosporine A; Aza, azathioprine, Eve, Everolimus. The data provided refer to the date of sample collection and inclusion in Multicenter study*.

### Biological Samples

Whole blood was collected in vacutainer serum tubes (BD, Becton Dickinson, USA) and serum was cryopreserved at −80°C until use.

### RNA Isolation

Total RNA was isolated from each serum sample (individually) using 1 ml of QIAzol solution added to 200 μl of serum. After incubation, 3.5 μl of the synthetic *miRNA*-39 from *Caenorhabditis elegans* (*cel-miR-39*; Qiagen, Germany) was added as a spike-in control, for data normalization. Then, 200 μl of chloroform (Merk, Germany) was added. The miRNeasy Serum/Plasma kit (Qiagen, Germany) was used for purification of cell-free total RNA, according to the manufacturer's instructions.

Serum quality was evaluated for each sample by determining the level of hemolysis by nanodrop 2,000 (nanoDrop Technologies, Delaware, USA). Samples displaying absorption peaks at 414 nm were withdrawn from the study due to free hemoglobin contamination (22).

### Complementary DNA Synthesis and Pre-amplification

Megaplex™ RT primers were used for cDNA synthesis. The Megaplex™ RT primers have two primer pools for human microRNAs, pool A, and pool B, each for reverse transcription of the microRNAs contained in the microfluidic cards. The experiment was performed according to the manufacturer's specification: 0.8 μL of Primers Megaplex™ RT (10×), 0.2 μL dNTPs with dTTP (100 mm), 1.5 μL of MultiScribe™ Reverse Transcriptase (50 U/μL), 0.8 μL of 10 × RT buffer, 0.9 μL of MgCl_2_ (25 mM), 0.1 μL RNase Inhibitor (20 U/μL), 0.2 μL Nuclease-free water (Applied Biosystems, Life Technologies, USA). For the microRNA profile after reverse transcription, a pre-amplification was required. This was completed following the criteria of the Kit containing TaqMan Pre Amp Master Mix (2x); Megaplex PreAmp Primers (10x); and water nuclease-free. For the *miR-885-5p* validation assays, we used: 3.5 μL of TaqMan Universal Master Mix II (containing 0.15 μL dNTPs, 0.5 μL of MultiScribe Reverse transcriptase, 3.5 μL of 10X RT buffer, 0.095 μl of RNAse Inhibitor) (Thermo Fisher Scientific, USA), 1.5 μL of the primer specific for *miR-885-5p* (5x), 2.5 μL of the RNA sample, and 2.08 μL of nuclease-free water.

### qPCR for *microRNA* Global Profile

Quantitative real-time PCR was performed using pre-printed TaqMan® Low Density Array (TLDA) microfluidic cards (Human Card A + B, each for 384 microRNAs; Applied Biosystems, CA, USA), with a panel for 768 microRNAs. Each card set contained MGB labeled probes specific for mature *miRNAs* and endogenous small nucleolar RNAs (for data normalization and relative quantification). The sample/master mix for each Megaplex pool was loaded into the cards, centrifuged and sealed with the Applied Biosystems sealer device.

Real time-PCR reactions were performed using QuantStudio 12K Flex (Thermo Fisher Scientific, USA) in individual serum samples. Raw TLDA data files were pre-processed with threshold and baseline corrections for each sample (automatic baseline and threshold set to 0.3) and each amplification plot was assessed to confirm that the cycle threshold (Ct) values corresponded to the midpoint of logarithmic amplification. The results are expressed in Fold Change (FC), which represents how many times a given microRNA is detected/expressed in one sample over another.

### qPCR for *miR-885-5p* Detection in Validation Assays in a Larger Number of Study Subjects

Complementary DNA was reverse transcribed using a Taqman *microRNA* Reverse Transcription kit (Thermo Fisher Scientific, USA). Briefly, 10 ng of the extracted total RNA was added to a mix containing 1.5 μL of 10x Reverse Transcription Buffer, 0.15 μL of 100 mM dNTP mix, 0.19 μL of RNase Inhibitor, and 4.16 μL of distilled-deionized water. Three microliters of specific RT-primers for hsa-*miR-885-5p* or *cel-miR-39* were used for directed cDNA synthesis. For the qPCR reactions 10 μL of TaqMan Universal Master Mix II at UNG (Thermo Fisher Scientific, USA) and 1 μL of specific Taqman *microRNA* Assay primer (hsa-*miR-885-5p* or *cel-miR-39*) were mixed to 1.33 μL of cDNA and 7.67 μL of distilled-deionized water. For greater reliability of the data, all reactions were performed as triplicates, for each serum sample, separately.

Real time-PCR reactions were performed using QuantStudio 12K Flex (Thermo Fisher Scientific, USA) thermocycler with the following cycling conditions: 95°C for 10 min, 40 cycles of 95°C for 15 s, and 60°C for 1 min. Data were analyzed using Excel 2010 (Microsoft, USA using the comparative calculation of Ct (2-ΔΔCt) (23). The results are expressed in Fold Change (FC), which represents how many times a given *microRNA* is detected/expressed in one sample over another*. Fold change* = *2-*${}^{\left[\left(CtmiR-\bar{X}Ctcontrol\right)SampleA-\left(CtmiR-\bar{X}Ctcontrol\right)SampleB\right]}$.

### Statistical and Data Pathway Analyses

For analysis of the levels of the 768 microRNAs, we used the web-based software (Cloud, Thermo Fisher® https://www.thermofisher.com/br/en/home/cloud.html). The differences between the groups are presented in volcano plots, using the unpaired Student's *t*-test, with previous data normalization. For *miR-885-5p* analysis, the results were plotted in graphs using Graphpad Prism 6.0 (Graphpad Software Inc., CA, USA) and statistical analyses were performed by the Kruskall-Wallis method, followed by the Dunns post-test for intergroup comparison. Differences are considered significant if *p* < 0.05.

### Gene Set Enrichment Analyses

Ingenuity Pathway Analysis (IPA) software (Qiagen) was used to identify targets of the differentially detected *microRNA*s in OT vs. CR. IPA target filter tool, based on the content of 01-2018 release, incorporates the TargetScan, TarBase, and miRecords algorithms and was used to find the list of highly predicted and experimentally validated targets of the differentially expressed *microRNA*s (*P* < 0.05 and absolute fold change FC >1.5) in each condition, considering the following features: microRNA, target scan human (database), high prediction, pathways involved, and no restriction of any biological context studied (such as transplantation or drug use). The idea was not to direct the analysis to tendentious results. Targets and pathways were free of restrictions. Based on the IPA database of molecular network interactions (Ingenuity Knowledge Base, IKB) we identified significantly enriched canonical pathways within the list of differentially expressed *microRNA*s targets (significance according to Fisher's exact test). In addition, we performed a thorough search in the literature, checking for additional experimentally demonstrated targets for the seven differentially detected *microRNA*s, present in these canonical pathways. In this additional search, we found that CASP3 has been experimentally shown do be a target for *miR-885-5p* (20) and BCL2, a reported target of miR-27a-5p (24). The *Search&Color Pathway* tool, from the Kegg Mapper platform (https://www.kegg.jp/kegg/tool/map_pathway2.html) was another strategy we used to map and color the list of mRNA targets on signaling pathways.

## Results

### Operational Tolerance Has a Differential microRNA Profile Compared to Chronic Rejection

**Figure 1:**
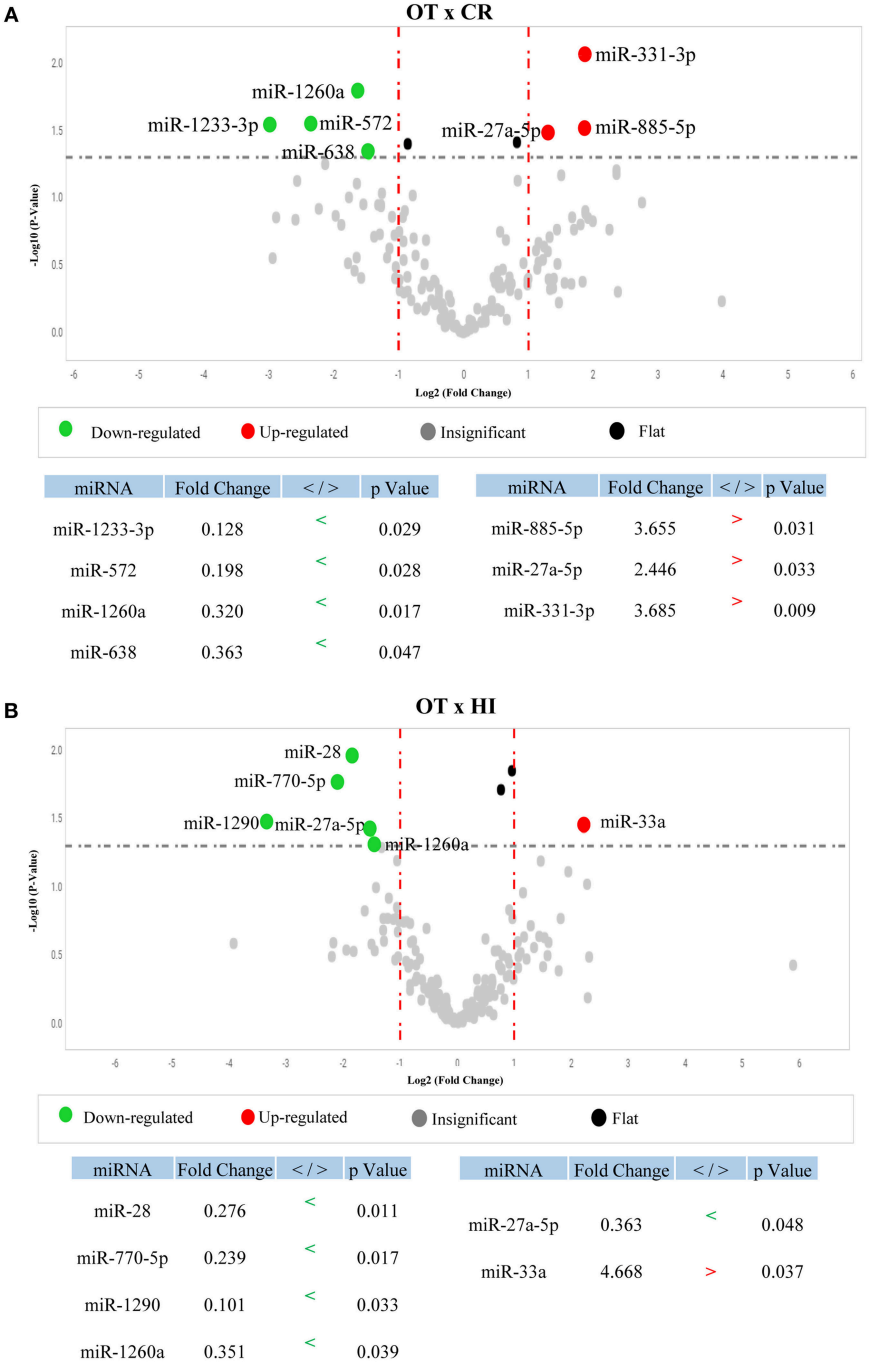
Differential profile of microRNA serum levels in Operational Tolerance. **(A)** Volcano plot shows microRNAs differentially detected between Operational Tolerance (OT) x Chronic Rejection (CR) (*OT-CR differential profile*). OT *n* = 8; CR *n* = 5. Red dots indicate microRNAs with higher levels and green dots indicate lower levels in OT in comparison to CR. The data are shown in fold change (x-axis) and statistical significance (–log10, *p*-value, y-axis). The dashed gray line indicates that the dots above have a *p* < 0.05 and the dots below the line a *p* > 0.05. Statistical calculation by *t*-test. **(B)** Volcano plot shows microRNAs differentially detected between Operational Tolerance (OT) x Healthy Individuals (HI). OT *n* = 8; HI *n* = 5. Red dots indicate microRNAs with higher levels and green dots indicate lower levels in OT in comparison to HI. The data are shown in fold change (x-axis) and statistical significance (–log10, *p*-value, y-axis). The dashed gray line indicates that the dots above have a *p* < 0.05 and the dots below the line a *p* > 0.05. Statistical calculation by *t*-test.

To determine whether OT displays a differential serum microRNA profile, we first compared microRNA levels between OT and its opposing clinical outcome, chronic rejection (CR), using individual samples. We identified seven microRNAs from the 768 microRNA-panel with differential detection between OT and CR (Figure 1A); three with higher levels in OT (red): *miR-885-5p* (*p* = 0.031), *miR-331-3p* (*p* = 0.009), *miR-27a-5p* (*p* = 0.033), and four with lower levels: *miR-572* (*p* = 0.028), *miR-638* (*p* = 0.047), *miR-1233-3p* (*p* = 0.029), *miR-1260a* (*p* = 0.017). This set of microRNAs that were differentially detected between OT and CR (*OT-CR differential profile*) was the basis for elaborating hypotheses regarding potential biological roles of microRNAs in the OT. Of these seven microRNAs, five had preserved levels in OT, in relation to the physiological state (no significant differences) (*miR-885-5p, miR-331-3p, miR-572, miR-638, miR-1233-3p*) and two were decreased (*miR-27a-5p*, p = 0.048; and *miR-1260a, p* = 0.039) (Figure 1B). In contrast, CR displayed more alterations in these microRNAs' levels compared to the physiological state.

The results showing the comparison of microRNA serum levels between OT vs. STA (Stable graft function individuals on conventional immunosupressants), and CR vs. HI are shown in Supplementary Figures 1 and 2, respectively. We chose to present them as a supplementary data because these data are not critical for the interpretations regarding the potential role of the microRNAs in the context of operational tolerance.

### *In silico* Identification of Potential Targets of the microRNAs in the *OT-CR differential profile* and Their Functional Grouping

We aimed to identify potential target mRNAs using the IPA-Ingenuity pathway analysis software. We considered targets classified as highly predicted and experimentally observed (Table 2) to generate hypotheses regarding the potential mechanisms that underlie operational tolerance that involves the microRNAs of the *OT-CR differential profile*. First, we checked the number of putative targets. *MiR-1260a*, whose levels were lower in OT vs. CR (by IPA analysis) had the highest numbers of targets (290 targets), followed by *miR-27a-5p* (165 targets). Only 4 targets were registered in IPA as experimentally observed, three of which are regulated by *miR-331-3p* and one by *miR-638*. We also found two other experimentally confirmed targets, one for *miR-885-5p* (20) and the other for *miR-271-5p* (24). After quantifying the number of potential targets of these microRNAs, we identified that 238 of the 833 (28.6%) highly predicted and/or experimentally observed targets are related to the immune system.

**Table 2:** Number of targets of the microRNAs comprised in the *OT* vs. *CR differential profile*.

**microRNA**	**Number of targets**	**Number of highly predicted and experimentally observed targets**	**Number of experimentally observed targets**
*miR-1260a*	1,190	290	–
*miR-27a-5p*	1,001	165	1
*miR-1233-3p*	733	136	–
*miR-331-3p*	666	133	3
*miR-638*	506	60	1
*miR-885-5p*	309	45	1
*miR-572*	205	37	–
Total	4,610	866	6

*This differential profile of microRNA levels, for the global profile, was obtained by quantitative real-time PCR using pre-printed TaqMan® Low Density Array (TLDA) microfluidic cards, containing a panel for 768 microRNAs. Using Ingenuity Pathway Analysis (IPA), we first determined the total number of putative targets, and the number of highly predicted and experimentally observed targets for the microRNAs of the OT-CR differential profile. The experimentally demonstrated targets are (considering data in the IPA and data from the literature): for miR-27a-5p (BCL2), for miR-331-3p (CDCA5, ERBB2, KIF23), for miR-885-5p (CASP3), and for miR-638 (CDK2). OT, Operational Tolerance (n = 8); CR, Chronic rejection, (n = 5)*.

### Gene Set Enrichment Analyses Indicate That the Regulation of the Death Signaling Pathway by the microRNAs in the *OT-CR differential profile* May Integrate Mechanisms in Operational Tolerance

**Figure 2:**
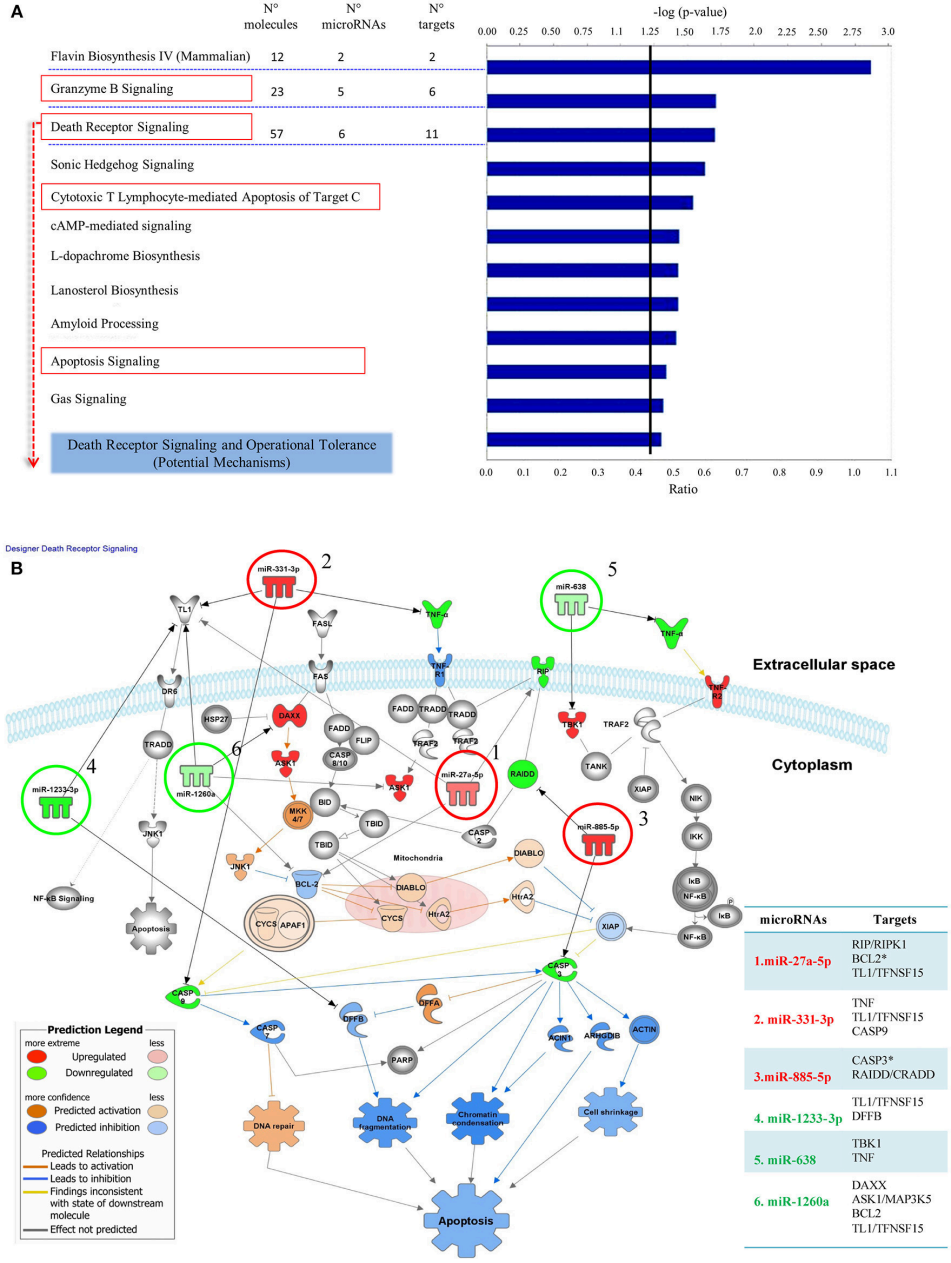
Signaling pathways comprising target molecules of microRNAs of the *OT-CR differential profile*. **(A)** The first 11 canonical pathways derived from the IPA (Ingenuity Pathway analysis), involving microRNAs of the *OT-CR differential profile*. Following the “Core Analysis” of the IPA analysis, these 11 canonical pathways arose. The chart shows category rankings. At the top of the graph we show the levels of significance (–log (*p*-value), Fisher's exact test, defined here at 1.25). The “Ratio” value represents the number of microRNA targets in the pathway divided by the total number of pathway molecules. N° molecules: total number of genes in the pathway; N° microRNAs: total number of the microRNAs of the OT-CR differential profile in the pathway; N° targets: total number of targets of the microRNAs comprised in the OT-CR differential profile, in the pathway. Boxes in red highlight the cell death pathways. **(B)** Graphical representation of the Death Receptor Pathway potentially regulated by the microRNAs of the OT-CR differential profile. The analyses are derived from the IPA software. In this pathway, six microRNAs of the differential profile may target several molecules involved in the mechanisms of cell death. The numbers in the figure indicate the miRNAs and the arrows link to their respective targets. (1) *miR-27a-5p* (targets: RIP/RIPK1, BCL2^*^,TL1/TFNSF15), (2) *miR-331-3p* (targets: *TNFTL1/TFNSF15, CASP9*), (3) *miR-885-5p* (targets: CASP3^*^, RAIDD/CRADD), (4) miR-1233-3p (targets: *TL1/TFNSF15, DFFB*), (5) miR-638: (targets: *TBK1,TNF*), (6) miR-1260a (targets: *DAXX, ASK1/MAP3K5, BCL2,TL1/TFNSF15*). The ^*^experimentally observed targets. The differences in microRNA expression are represented by the gradation of color intensity (hypoexpressed green and hyperexpressed red). The legend in the figure discriminates the prediction of the regulation of the targets. On the right side of the table we have the list of targets and the respective microRNAs involved in the pathway. ^*^Experimentally demonstrated targets.

We used Ingenuity Pathway Analysis (IPA) to identify enriched signaling pathways comprising targets regulated by microRNAs in the *OT-CR differential profile*. The idea was to explore how targets of these microRNAs may integrate the identified signaling pathways, and to determine potential mechanisms involved in operational tolerance. The pathways predicted to be regulated by the microRNAs integrating the *OT-CR differential profile* have functions, predominantly, in cell death processes (Figure 2A), namely the granzyme B, death receptor signaling, cytotoxic T lymphocyte-mediated apoptosis of target cells and apoptosis signaling, and in processes related to tissue maintenance/regeneration, such as the *sonic hedgehog* signaling pathway (Figure 2A). The IPA results revealed that six of the seven microRNAs of the *OT-CR differential profile* target several molecules within the death receptor pathway (Figure 2B). These six microRNAs of the *OT-CR differential profile* (*miR-1260a, miR-1233-3p, miR-638, miR-885-5p, miR-27a-5p, miR-331-3p*) presented together, several target genes that are key players in death signaling (e.g., RIPK1, BCL2, TL1, TNF, CASP3, CASP8, CASP9, CRADD, DAX, MAP3K5, DFFB, APAF1, and ATM), three shared targets (BCL2, TL1, TNF) for different microRNAs (Figure 2B). As summarized in the Table 3, the microRNAs *miR-885-5p, miR-27a-5p, miR-331-3p* could impact programmed cell death by downregulating the target molecules CASP3, CASP9, and RIPK1, contrasting with CR.

**Table 3:** Potential effects of the microRNAs comprised in the *OT-CR differential profile* on the different targets of the death receptor pathway.

***microRNA***	**Level of microRNA in OT vs. CR**	**Targets**	**Potential effects of the microRNA on targets OT x CR**	**Function of target molecules**	**Potential differential microRNA effects in OT vs. CR**
*miR-27a-5p*	OT > CR	*RIPK1* (Receptor interacting serine/threonine kinase 1)	<*RPK*1 (OT) >*RPK1* (CR)	Kinase involved in the transduction of necroptosis signals (25)	Decreased death stimulus in OT than in CR
		BCL2^*^	<*BCL2* (OT) >*BCL2* (CR)	Positive cell survival regulator (26)	Increased cell survival in CR
		*TL1 (TNFSF15) TNF* superfamily member 15	<*TNFS15*(OT) >*TNFS15*(CR)	Can activate *NF-kB* (27)	Lower expression of pro-inflammatory and cell survival genes in OT
*miR-331-3p*	OT > CR	*TL1 (TNFSF15)* (TNF superfamily member 15)	<*TNFS15*(OT) >*TNFS15*(CR)	Can activate *NF-kB* (27)	Lower expression of pro-inflammatory and cell survival genes in OT
		*TNF* (tumor necrosis factor)	<*TNF* (OT) >*TNF* (RC)	Involved in several proinflammatory responses (27)	Decreased proinflammatory responses in OT
		*CASP9* (Caspase 9)	<*CASP9*(OT) >*CASP9*(CR)	Activates *CASP3* (28)	Decreased cell death in OT than in CR
*miR-885-5p*	OT > CR	*CRADD/RAIDD* (*CASP2* and *RIPK1* domain containing adaptor with death domain)	<*CRADD*(TO) >*CRADD*(CR)	Induces DNA fragmentation (29)	Decreased cell death in OT than in CR
		CASP3^*^ (Caspase 3)	<*CASP3* (OT) >*CASP3*(CR)	Induces DNA fragmentation and condensation (30)	Decreased cell death in OT than in CR
*miR-1260a*	OT < CR	*TL1 (TNFSF15) TNF* superfamily member 15	>*TNFS15* (OT) < *TNFS15*(CR)	Can activate *NF-kB* (27)	Higher expression of pro-inflammatory and cell survival genes in OT
		*DAXX* (Death domain associated protein)	>*DAXX* (OT) < *DAXX* (CR)	Interacts with *FAS* that is involved in promoting apoptosis (31)	Induction of apoptosis in OT
		*MAP3K5 (ASK1)* Mitogen-activated protein kinase kinase kinase 5	>*MAP3K5*(OT) < *MAP3K5*(CR)	Activates c-Jun N-terminal kinase (JNK) and p38 mitogen-activated protein kinases (32)	Increased propagation of the signals of the MAP3K5 pathway in OT
		*BCL2*	>*BCL2* (OT) < *BCL2* (CR)	Positive cell survival regulator (26)	Increased cell survival in OT
*miR-638*	OT < CR	*TNF* (tumor necrosis factor)	>*TNF* (OT) < *TNF* (CR)	Involved in several proinflammatory responses (27)	Increased proinflammatory responses in OT
		*TBK1* (TANK-binding kinase 1)	>*TBK1* (OT) < *TBK1* (RC)	Can activate *NF-kB* (33)	Higher expression of pro-inflammatory and cell survival genes in OT
*miR-1233-3p*	OT < CR	*TL1 (TNFSF15*) *TNF* superfamily member 15	>*TNFS15*(OT) < *TNFS15*(RC)	Can activate *NF-kB* (27)	Higher expression of pro-inflammatory and cell survival genes in OT
		*DFFB* (DNA fragmentation factor subunit beta)	>*DFFB* (OT) < *DFFB* (RC)	Involved in promoting cell death (34)	Increased cell death in OT than in CR

*(>) higher; (< ) lower, comparing OT vs. CR. OT, Operational Tolerance; CR, Chronic rejection*.

* *Targets experimentally observed in cell death pathways*.

### Higher Levels of *miR-885-5p* in OT May Be Implicated in CRADD/RAIDD and CASP3 Decreases and the Down Regulation of Cell Death

**Figure 3:**
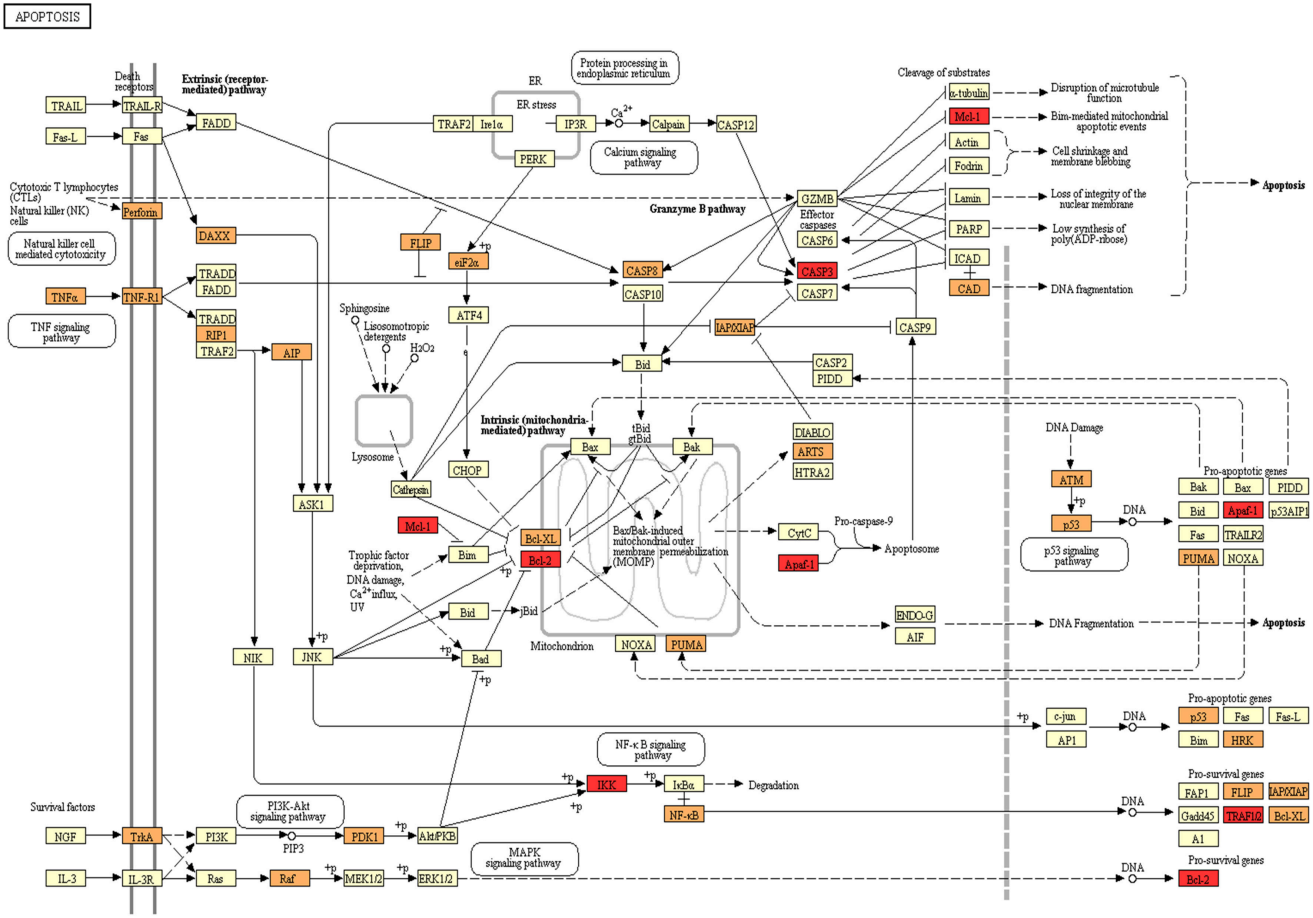
Programmed cell death as a target for microRNAs in OT. Kegg Mapper webtool was used to map the influence of the OT-CR differential microRNA profile. Members of Apoptosis pathway annotated in Kegg are shown in yellow. Genes targeted by differentially detected microRNAs in OT are shown in orange. Targets of miR-885-5p are marked in red (Mcl-1; Bcl-2; Apaf-1, CASP3, IKK, TRAFIQ).

**Figure 4:**
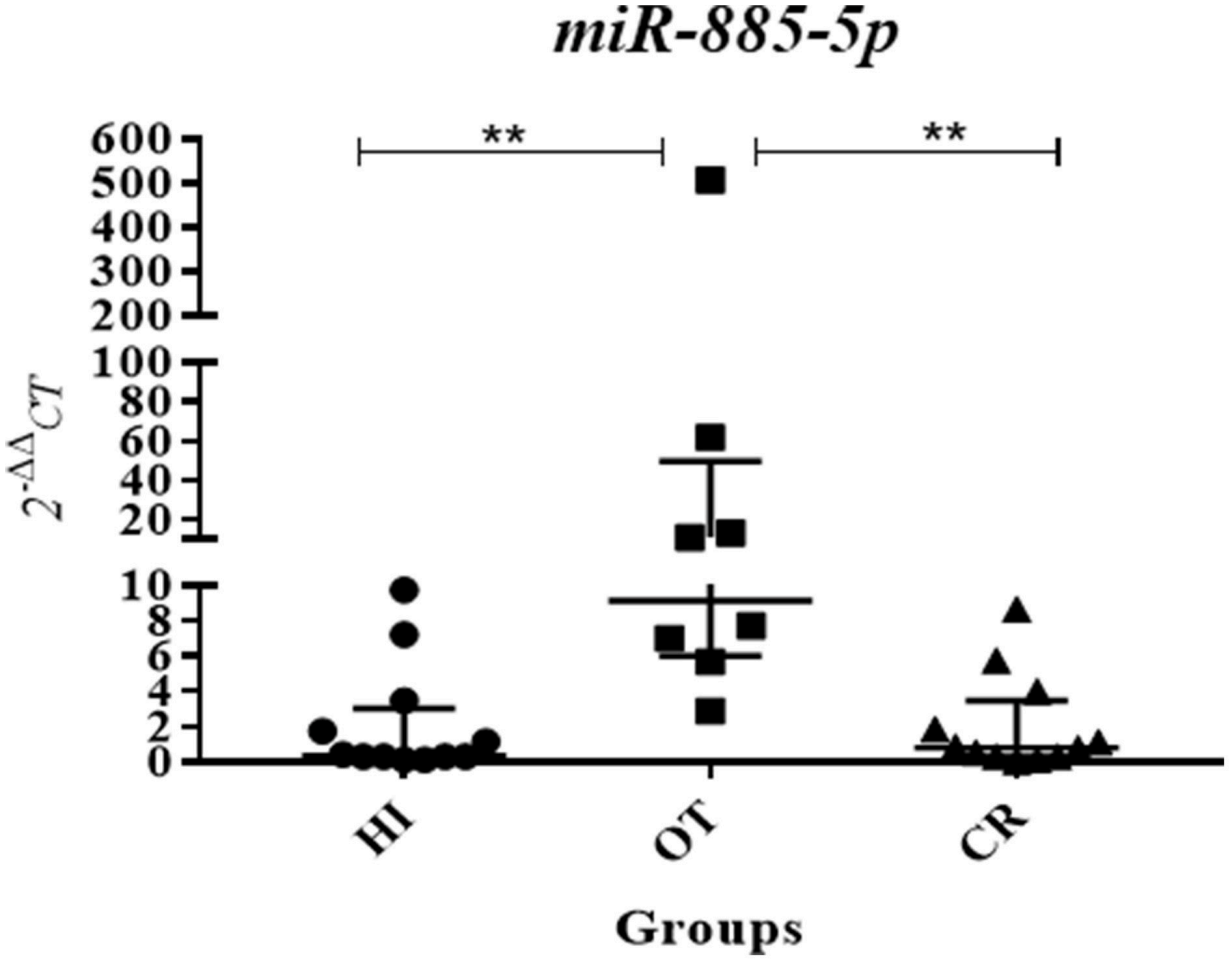
Validation assay showing increased levels of *miR-885-5p* in operational tolerance. Following the detection of higher levels of *miR-885-5p* in OT compared to chronic rejection in the global analysis, we performed a validation experiment by individual quantitative real-time PCR, using a specific primer for *miR-885-5p*, in a larger number of study subjects, using individual samples (OT *n* = 8, CR *n* = 12, HI *n* = 12; OT, operational tolerance; CR, chronic rejection; HI, Healthy individuals). Values are expressed as 2^−^ΔΔCt for *miR-885-5p* serum levels. For the statistical calculation we used the Kruskal-Wallis method followed by the Dunns post-test, and the results were considered significant if *p* < 0.05. ^**^p = 0.0063 (OT × CR) and 0.0035 (OT × HI).

**Figure 5:**
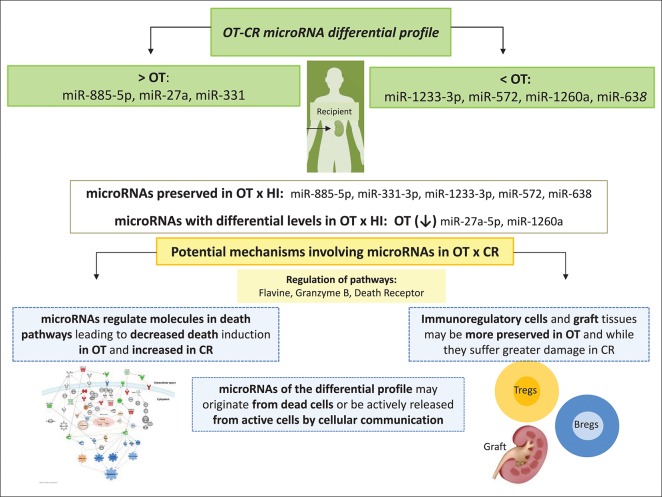
Summary of our main findings and some hypotheses we have raised for potential mechanisms in OT, involving the microRNAs comprised in the *OT-CR differential profile*. At the top of the figure, we show the set of microRNAs comprised in the *OT-CR differential profile* indicating those presenting higher or lower levels in OT. Then, we indicate the microRNAs whose levels may be preserved in OT in relation the physiologic state, and those with decreased levels. Among the potential mechanisms of tolerance involving these microRNAs, we highlight the death receptor pathway. Our hypothesis is that OT may also involve mechanisms mediated by microRNAs affecting the cell death pathway. These mechanisms would favor the survival of cells most likely with the regulatory profile, as well as of graft tissue, contrary to what is could be occurring in CR. Understanding these mechanisms may contribute to the development of novel strategies to promote cell survival in renal transplantation, favoring the development of transplantation tolerance, in the future.

We selected *miR-885-5p* to validate microRNA levels in a larger number of subjects (OT: *n* = 8, CR: *n* = 12, HI: *n* = 12). This selection was based on the fact that the IPA analysis for *miR-885-5p* showed predicted targets that are critical in various biological processes, including key targets in cell death pathways (Figure 3). In particular, *miR-885-5p* was predicted to target crucial members in death signaling, such as BCL2, MAPK1, CTSC, IKBKB, MCL1, APAF1, CASP3, NRAS, and TRAF1. We confirmed the higher *miR-885-5p* levels in OT vs. CR (*p* = 0.0063) and now, also higher than in healthy individuals (*p* = 0.0035) (Figure 4). This offers an interesting opportunity to generate novel and potentially relevant data, on human transplantation tolerance. To summarize our main findings, we present some hypotheses we have raised for potential mechanisms in OT that involve the microRNAs comprised in the *OT-CR differential profile*. It is worth noting whether targets have been experimentally demonstrated or are highly predicted (Figure 5).

## Discussion

We found a differential profile of microRNA levels in operational tolerance, in relation to its opposing clinical outcome—chronic rejection. These data suggest that microRNAs may integrate the network of mechanisms involved in human transplantation tolerance. In the *OT-CR differential profile*, OT presented higher levels than CR, for three microRNAs (*miR-27a-5p, miR-331-3p, miR-885-5p*) and lower for four (*miR-638, miR-572, miR-1260a, miR-1233-3p*). The differential microRNA profile between OT and CR occurred primarily due to alterations in CR in relation to the physiological state, indicating that in OT these microRNA levels were mostly preserved, which does not occur in chronic rejection.

The gene set enrichment analysis (GSEA) using the *Ingenuity Pathway Analysis* (IPA) platform showed that the predicted mRNA targets for the *OT-CR differential microRNA profile* strongly impact signaling pathways and biological processes related to programmed cell death and survival, such as the Death Receptor Signaling and Cytotoxic T Lymphocyte-mediated Apoptosis of target cells. We chose the death receptor pathway to explore the potential differential effects of the microRNAs, because it emerged as a predominant pathway that potentially affects six of the seven microRNAs (Figure 2B and Table 3). Moreover, although regulation of cell death is widely accepted to be crucial in maintaining homeostasis, it has not been deeply investigated in the context of operational tolerance.

The targeting of crucial members of Apoptosis signaling and/or the immune system lead us to investigate *miR-885-5p* serum levels in a larger number of subjects (Figure 4). In addition, Caspase 3, which is critical in inducing apoptosis, is an experimentally demonstrated target for *miR-885-5p* (20). Our validation assays confirmed that there were higher levels in OT vs. CR and vs. the physiological state, indicating the occurrence of systemic *miR-885-5p* upregulation in OT that contrasts to decreased levels found in CR.

The increased *miR-885-5p* levels in OT could lead to down regulation of both CRADD/RAIDD (a molecule bearing CASP2 and RIPK1 domain containing adaptor with death domain) and CASP3 expression, which favors that *miR-885-5p* contributes to the control of cell death. In contrast, the lower *miR-885-5p* levels in CR would lead to higher CASP3 and CRADD/RAIDD expression, and favor the execution of the cell death program. It is important to mention that it is not known which specific cell types differentially express *miR-885-5p* in OT vs. CR. Nevertheless, considering these opposing clinical outcomes—tolerance or chronic rejection—we hypothesize that *miR-885-5p* has actions in OT and may promote and/or be related to cell survival in immune cells bearing immunoregulatory activities while, in CR, it would rather favor cell death of immunoregulatory lymphocytes.

In line with this interpretation, our group and others have shown that OT preserves regulatory T (4, 5) and B cell (6, 7) numbers similarly to healthy individuals, in contrast to CR subjects who have decreased regulatory lymphocytes numbers. The mechanisms involved in regulatory lymphocyte number preservation in OT (and loss in CR) require further investigation. It is possible that there is Treg exhaustion (35) in CR, or that the deleterious effects of immunosuppressants such as calcineurin inhibitors favor Treg death (36), and other non-exclusive mechanisms. Considering that our current data show differential miRNA levels and their putative impact on the cell death pathway, we propose that, together with other potential mechanisms, these miRNAs could play a relevant role in regulating the death of several blood cells, including regulatory T and B cells, which favor survival in OT and death in CR. This highlights the participation of epigenetic mechanisms in operational tolerance. Although mTOR inhibitors such as rapamycin/everolimus preserve Treg function (36), this favorable effect is improbable in our study, because only two individuals used such immunosupressants; one in the CR group and another in the OT group (Table 1). This interpretation is supported by the fact that *miR-885-5p* has been experimentally demonstrated to target *CASP3* (20), decreasing *CASP3* mRNA and protein expression and leading to the evasion of cell death (20). This study involved cells from cancer subjects and used luciferase reporter assays. We hypothesize that a similar phenomenon may be occurring in operational tolerance, but in regulatory lymphocytes and not only mediated by *miR-885-5p* and their targets, but possibly also by other molecules that are targeted to the other miRNAs of the *OT-CR differential profile* (summarized in Table 3). It is also notable that although the other targets are (to date) classified as highly predicted, if these are confirmed as true targets, the overall outcome of their various actions would predominantly favor cell survival. We only used *miR-885-5p* and CASP3 as an example for discussion, because it has been experimentally demonstrated as a target.

In addition to acting on caspase 3, in the Death Receptor pathway, other potential actions on other target molecules could be discussed. We should mention, however, that these have not been experimentally demonstrated as targets. For instance, *miR-885-5p* may regulate the *RAIDD/CRADD* complex (*CASP2* and *RIPK1* domain containing adapter with death domain—*CRADD*) (29). *CRADD* recruits caspase 2 to the cell death signal transduction complex, including the death receptor (*TNFR1A*) and the *RIPK1/RIP* kinase (interacting serine/threonine kinase 1 receptor), promoting death; once again, favoring less cell death in OT and more in CR. For the other *microRNA*s in the *OT-CR differential profile* there are some potentially relevant activities that favor tolerance, which opposes to CR. For example, *miR-331-3p* can regulate *CASP9*, which activates *CASP3* (28) and leads to cell death. Thus, the higher levels of this microRNA in OT may be related to or promote downregulation of *CASP9* and consequently, less *CASP3* activation, which favors cell survival in OT and cell death in CR. *MiR-27a-5p*, whose levels were also higher in OT, could limit cell death, by acting on *RIPK1* (a receptor interacting serine/threonine kinase 1), a molecule involved in necroptosis cell death, a caspase-independent programmed cell death with a dominant inflammatory outcome (25, 37). The downregulation of *RIPK1* would be related to (or promote) the decreased occurrence of death with an inflammatory outcome in OT. All *microRNA*s in the *OT-CR differential profile*, display actions that potentially affect cell death/survival in a differential manner in OT and CR, and the overall outcome seems to favor survival in OT (Table 3).

An important issue to discuss is the biological significance of serum microRNA, and if it can be a readout for intracellular microRNA functional activities. Published data show that microRNAs are abundant and stable in the serum and that indeed serum microRNAs may indicate ongoing intracellular activities (38). Thus, it seems reasonable to interpret that the microRNAs differentially detected in OT in relation to CR could indicate ongoing mechanisms that favor tolerance or chronic rejection. In the biological contexts of our study, we should mention that the detected serum microRNAs may also be derived from the graft itself. The graft microenvironments in OT and CR are very different: predominantly immunoregulatory in OT and inflammatory in CR. Accordingly, the type of cell death taking place in these opposing graft contexts probably differs, most likely mediated by apoptosis in OT and necrosis/necroptosis in CR. However, apoptosis vs. necroptosis remains unexplored in operational tolerance. Another issue for future investigation is whether the differential microRNA serum levels could also be implicated in a differential source of active molecules that could access the intracellular compartments and differentially affect cell functions, by downregulating mRNA and protein expression. Given that exosomes do enter intracellular compartments (13, 39) and that microRNAs can be coupled to exosomes (14, 15), this is quite conceivable.

We believe that, together with several other mechanisms, epigenetic mechanisms contribute to cellular decisions and cell fate, mounting the systemic possibility of supporting homeostasis after transplantation. Certainly, an important issue to consider is whether the differential levels of microRNA in OT are caused by or consequences of tolerance. Either way, given that these microRNAs display significant differences in relation to OT's opposing clinical outcome, a reasonable interpretation is that their ongoing actions have distinct ongoing effects. Only with the further accumulation of data that may give support to either direction will help the scientific community to have a more robust interpretation. Nevertheless, we believe that the debate is enriched by raising the hypothesis that epigenetic mechanisms such as those promoted by microRNAs may have a relevant biological impact and contribute to the outcomes of tolerance or chronic rejection.

## Conclusion

The existence of a differential profile in the systemic levels of miRNAs in the operational tolerance, in relation its opposing clinical outcome (chronic rejection) suggests that miRNAs may participate in the mechanisms involved in immune tolerance in human transplantation. These *microRNA*s are potentially involved in different signaling pathways that are mostly related to cell death, suggesting that the regulation of cell death pathways can integrate the underlying mechanisms in operational tolerance by regulating caspases and molecules related to cell survival. The higher levels of *miR-885-5p* in operational tolerance in relation to CR and to the physiological state in healthy individuals suggest that this microRNA integrates the network of transplantation tolerance mechanisms. We propose that the complex and dynamic immunobiology of human allograft tolerance also involves epigenetic mechanisms, which include the control of molecules that mediate cell survival and death. Determining critical mechanisms involved in the life/death of regulatory lymphocytes, for instance, may be instrumental for developing novel therapeutic strategies to control/prevent cell death and favor tolerance.

## Ethics Statement

This study was approved by ethic committee at the School of Medicine-USP (CAPPesq-Ethics Committee for Research Projects; 0476/08 and 1097/05). All study subjects signed an informed consent.

## Author Contributions

AC: contribution to the conception of the work, data acquisition, analysis and interpretation of data, wrote the manuscript, critically revising. DdSC: contribution to the conception of the work, data acquisition, analysis and interpretation of data, critically revising. SM: contribution to the analysis of data and final approval of the version to be published. FL, DS, and IN: contribution to the analysis of data and discussion of clinical issues, critically revising. LA: contribution to the data acquisition and analysis of data. MG: contribution to the analysis of data and critical revision of the manuscript. JK: discussion of clinical issues, critically revising. EC-N: contribution to the conception and design of the work, contribution to the analysis of data, critically revising. LF: contribution to the conception and design of the work, contribution to the analysis of data, critically. VC: mentor; substantial contributions to the conception, design, analysis and interpretation of the work, wrote the manuscript, critically. All authors read and approved the final manuscript.

### Conflict of Interest Statement

The authors declare that the research was conducted in the absence of any commercial or financial relationships that could be construed as a potential conflict of interest.

## Supplementary Material

Supplementary Figure 1 Differential profile of microRNA serum levels in OT × STA. Volcano plot shows microRNAs differentially detected between Operational Tolerance (OT) vs. Stable graft function individuals on conventional immunosupressants (STA) (OT-STA differential profile). OT *n* = 8; STA *n* = 4. Red dots indicate microRNAs with higher levels and green dots indicate lower levels in OT in comparison to STA. The data are shown in fold change (x-axis) and statistical significance (−log10, *p*-value, y-axis). The dashed gray line indicates that the dots above have a *p* < 0.05 and the dots below the line a *p* > 0.05. Statistical calculation by *t*-test.

Supplementary Figure 2 Differential profile of serum microRNA levels in CR × HI. Volcano plot shows microRNAs differentially detected between Chronic rejection (CR) vs. Healthy individuals (HI) (CR-HI differential profile). CR *n* = 5; HI *n* = 5. Red dots indicate microRNAs with higher levels and green dots indicate lower levels in CR in comparison to HI. The data are shown in fold change (x-axis) and statistical significance (−log10, *p*-value, y-axis). The dashed gray line indicates that the dots above have a *p* < 0.05 and the dots below the line a *p* > 0.05. Statistical calculation by *t*-test.

## Abbreviations

CR: Chronic rejection
OT: Operational Tolerance
HI: Healthy individuals
*microRNA*s or miR: MicroRNAs
PBMC: Peripheral blood mononuclear cell
*CASP3*: Caspase 3
TLDA: TaqMan® Low Density Array
qPCR: Quantitative real-time
IPA: Ingenuity Pathway Analysis
mTOR: The mammalian target of rapamycin
Treg: Regulatory T cell
*RIPK1*: Receptor interacting serine/threonine kinase 1
*TL1 (TNFSF15)*: *TNF* superfamily member 15
*TNF*: Tumor necrosis factor
*CASP9*: Caspase 9
*CRADD/RAIDD*: CASP2 and RIPK1 domain containing adaptor with death domain
*DAXX*: Death domain associated protein
*MAP3K (ASK1)*: Mitogen-activated protein kinase kinase kinase 5
*TBK1*: TANK-binding kinase 1.
