# Supplementary figures and images for: Differential microRNA Profile in Operational Tolerance: A Potential Role in Favoring Cell Survival
Source: Front Immunol. 2019 Apr 25;10:740. doi: 10.3389/fimmu.2019.00740 (PMC6496457; doi:10.3389/fimmu.2019.00740)

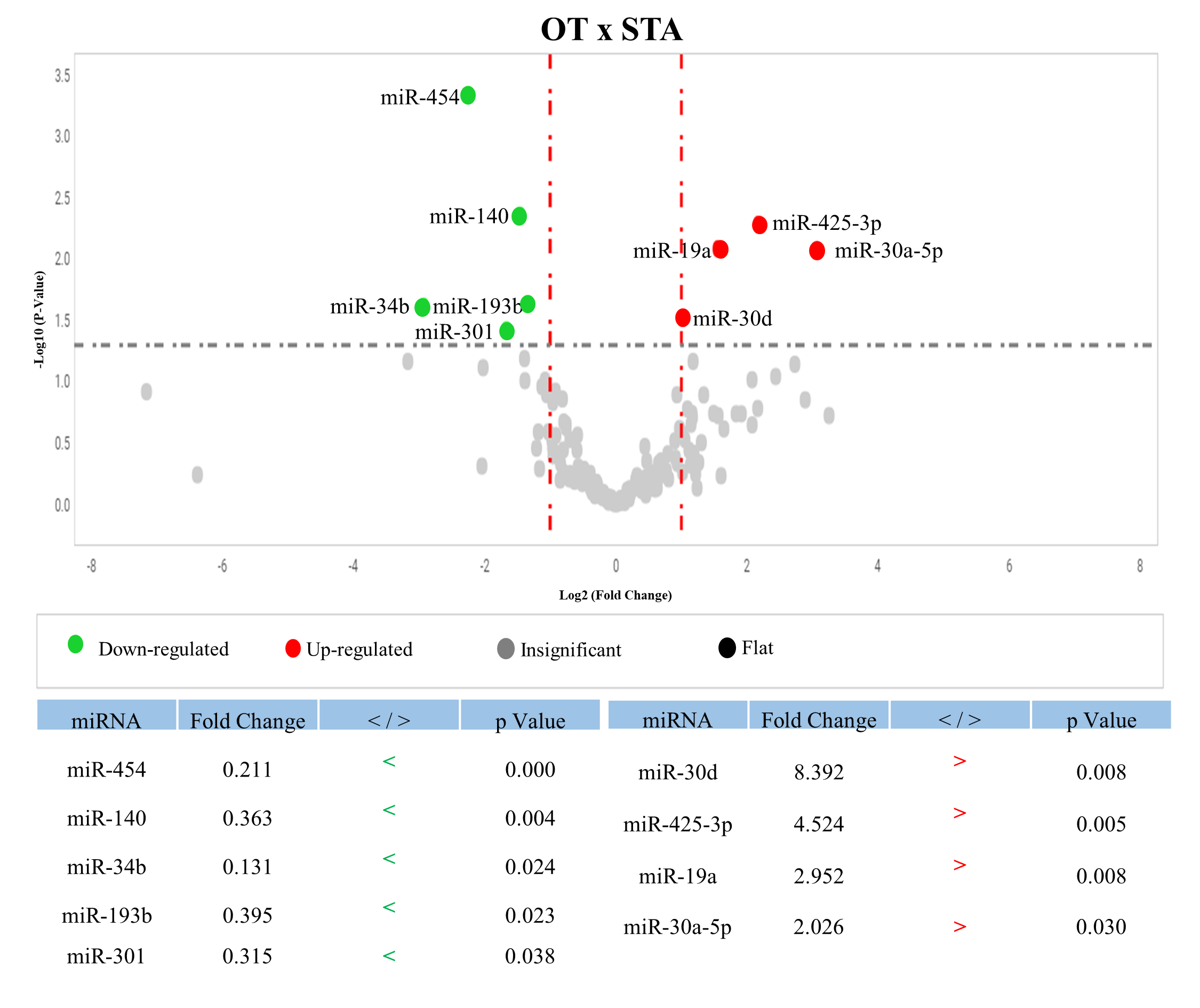

Supplement: Supplementary Figure 1 — Differential profile of microRNA serum levels in OT × STA. Volcano plot shows microRNAs differentially detected between Operational Tolerance (OT) vs. Stable graft function individuals on conventional immunosupressants (STA) (OT-STA differential profile). OT n = 8; STA n = 4. Red dots indicate microRNAs with higher levels and green dots indicate lower levels in OT in comparison to STA. The data are shown in fold change (x-axis) and statistical significance (−log10, p-value, y-axis). The dashed gray line indicates that the dots above have a p < 0.05 and the dots below the line a p > 0.05. Statistical calculation by t-test. [file Image_1.tif]

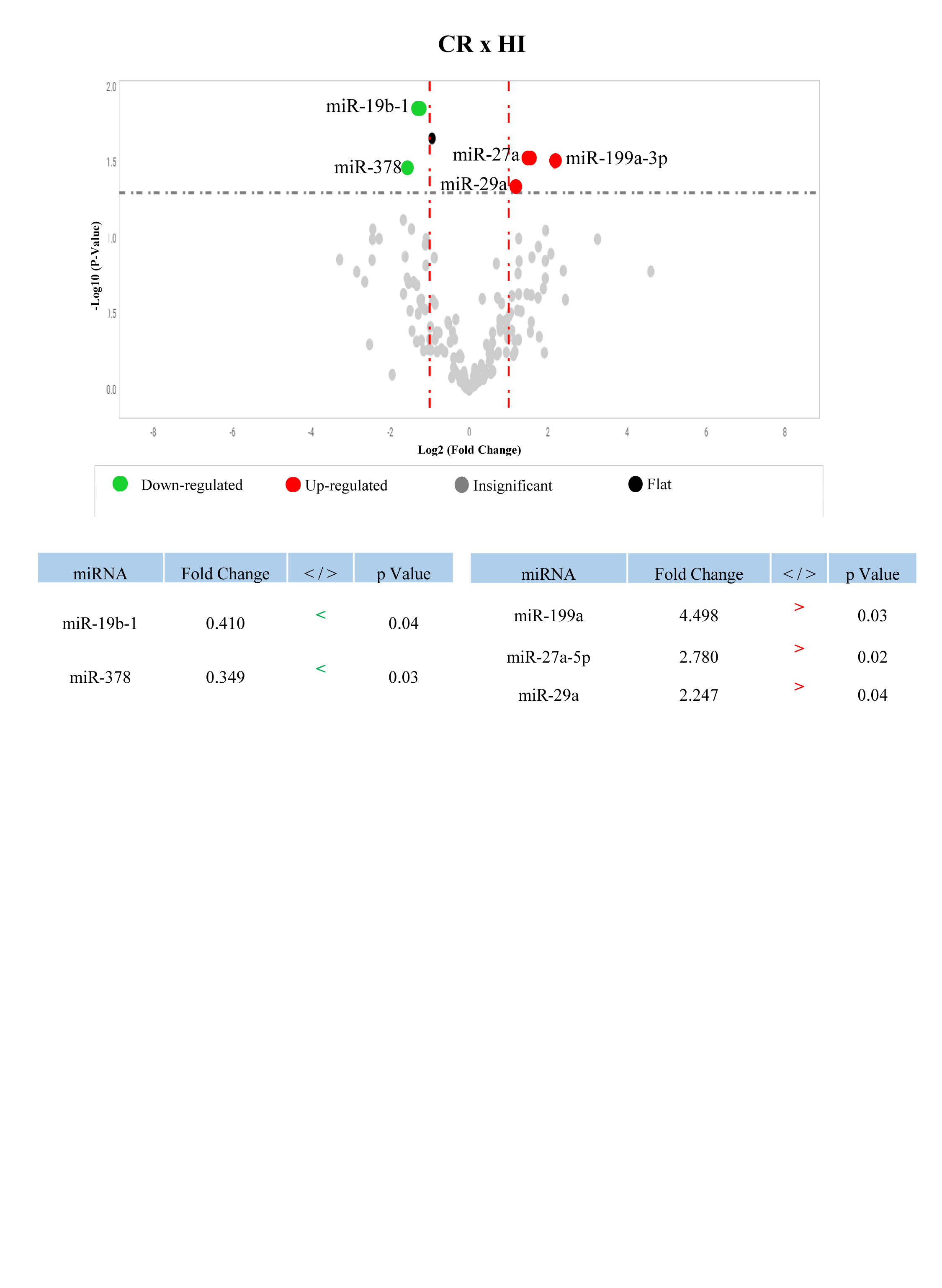

Supplement: Supplementary Figure 2 — Differential profile of serum microRNA levels in CR × HI. Volcano plot shows microRNAs differentially detected between Chronic rejection (CR) vs. Healthy individuals (HI) (CR-HI differential profile). CR n = 5; HI n = 5. Red dots indicate microRNAs with higher levels and green dots indicate lower levels in CR in comparison to HI. The data are shown in fold change (x-axis) and statistical significance (−log10, p-value, y-axis). The dashed gray line indicates that the dots above have a p < 0.05 and the dots below the line a p > 0.05. Statistical calculation by t-test. [file Image_2.tif]
